# Multidimensional Intelligences Model as a predictor for coming out acceptance and growth among LGB non-migrant Hispanics: A pilot study

**DOI:** 10.21203/rs.3.rs-2383001/v1

**Published:** 2023-02-08

**Authors:** Caleb Esteban, Alixida Ramos-Pibernus, Juan A. González-Rivera, Edna Mattei-Torres, Eddiel Hernández-López

**Affiliations:** Ponce Health Sciences University; Ponce Health Sciences University; Ponce Health Sciences University; Ponce Health Sciences University; Ponce Health Sciences University

**Keywords:** LGB/LGBT, Sexual Minorities, Coming Out Acceptance, Coming Out Growth, Fluid Intelligences

## Abstract

The coming out process has biopsychosocial components that occur whenever a lesbian, gay or bisexual (LGB) person shares their sexual orientation with another person. It is a complex and difficult process, but it has been described as an essential component for identity formation and integration. The purpose of this pilot study was to assess a Multidimensional Intelligences Model (MIM) (Body, Emotional, Social, and Ecological Intelligences) as predictor of the coming out acceptance (COA) and growth (COG).The objectives of this study were to 1) examine if MIM can predict COA and COG among LGB individuals; and 2) determine differences in the MIM between the four stratified groups (lesbian women, bisexual women, gay men, bisexual men). This pilot study had a quantitative method with an exploratory cross-sectional design. A total of 67 LGB participants completed an online survey. The results shows that the MIM could not predict the COA. However, the MIM could predict the COG and explained 20.6% of the variance. We believe this can be explained because in the COA people do not have control of how other people can feel and react. However, in the COG the person could have full self-control of their own growth.

## Resumen

El proceso de salir del clóset tiene componentes biopsicosociales que ocurren cuando una persona lesbiana, gay o bisexual (LGB) comparte su orientación sexual con otra persona. Es un proceso complejo y difícil, pero ha sido descrito como un componente esencial para la formación e integración de la identidad. El propósito de este estudio piloto fue evaluar un Modelo de Inteligencias Multidimensionales (MIM) (Inteligencia Corporal, Emocional, Social y Ecológica) como predictor de la aceptación después de la salida del clóset (ASC) y el crecimiento después de la salida del clóset (CSC). Los objetivos de este estudio fueron 1) examinar si MIM puede predecir el ASC y el CSC entre personas LGB; y 2) determinar diferencias en el MIM entre los cuatro grupos estratificados (mujeres lesbianas, mujeres bisexuales, hombres gays, hombres bisexuales). Este estudio piloto contó con un método cuantitativo con un diseño transversal exploratorio. Un total de 67 participantes LGB completaron una encuesta en línea. Los resultados muestran que el MIM no predice la ASC. Sin embargo, el MIM sí predice el CSC, explicando el 20,6% de la varianza. Creemos que esto se puede explicar porque en la ASC las personas no tienen control de cómo otras personas pueden sentir y reaccionar. Sin embargo, en la CSC la persona puede controlar su propio crecimiento.

## Introduction

Scientific evidence sustains that in the coming out process, a more Coming Out Acceptance and Coming Out Growth are related to better mental health and wellbeing [1, 2, 3]The coming out process, also known as the disclosure of the sexual orientation, is a life process that sexual minorities such as Lesbian, Gay, or Bisexual (LGB) persons face, and it is faced differently according to the sex, gender, sexual orientation, and other identities of the person [4].

The coming out process has biopsychosocial components (e.g., chronic stress, anxiety, family rejection) that occur whenever an LGB person shares their sexual orientation with another person. It is a complex and difficult process, but it has been described as an essential component for identity formation and integration [1, 5]. The successful coming out to others can lead to mental health benefits, contrarily negative consequences can lead to stressors like family, friends and acquaintances’ rejection, discrimination, prejudices, victimization, violence, and harassment [6, 7, 8]. Literature suggests that those stressors can be doubled in individuals who do not have social support [9, 10].

Most individuals experience disproportionate sensations of fear, guilt, confusion, doubt, denial, and religious/spiritual internal conflicts during this process which is not only once in life but can be extended throughout the lifespan [4, 11, 12]. The positive effects of coming out are linked to better mental health, wellbeing, higher self-esteem, lower anxiety, emotional relief, work satisfaction, increased coping resources, and resilience [1, 13, 14]. The negative consequences of the coming out can positively change with the development of programs and psychoeducational interventions to plan, prepare, and support the coming out process [15, 16]

On the other hand, fluid intelligences are those that we learn on the path of our life and help us solve more effectively our difficulties and evaluate various solutions [17]. Therefore, fluid intelligences can be integrated into biological, psychological, social, and ecological dimensions. Therefore, we created the Multidimensional Intelligences Model (MIM) that can help conceptualize and promote healthier coming out process. Based on a biopsychosocial-ecological approach, we connected the following intelligences instrument to address all four dimensions: Body Intelligence (Biological Level) [18] Emotional Intelligence (Psychological Level) [19] Social Intelligence (Social Level) [20], and Ecological Intelligence (Ecological Level]) [21].

Body Intelligence assesses forms of body awareness that support overall wellness. It is the awareness and use of bodily sensations that we use to support health and well-being, supply information about environmental safety and comfort, and enhance personal and spiritual development to support overall health and well-being [18]. On the other hand, emotional intelligence assesses a form of relational intelligence that involves the ability to monitor the self, our own feelings and emotions, discriminate between them, and use the information to guide thoughts and actions [19]. Furthermore, social intelligence assesses social understanding and interest, as well as the ability to interpret social information leading to accurate social inferences. This construct includes cognitive and behavioral sociocultural competencies such as social information processing, social skills, and social awareness [20]. Finally, ecological intelligence assesses the systemic thinking, ecophilosophy, holistic perspective, collective lifestyle, and cultural commons. Ecological intelligence is the capacity to learn from experiences and deal effectively with our environment, understanding of organisms and their ecosystems [21].

In the Hispanic culture, a collectivist culture, there is particular importance on close family relationships titled familism. Hispanics place greater emphasis on family relationships as their priority to obtain social support, making the acceptance of the coming out process extremely important in the Hispanic culture [22, 23]. When Hispanics experience coming out rejection from their family, it can be perceived as discrimination, prejudice, victimization, and/or harassment among others. Studies of young, gay Hispanics in Puerto Rico reports that only 23% of their mothers and 20% of their fathers accepted them during their coming out process, while their parents were also the people that most frequently rejected them because of their sexual orientation [24].

Emotional intelligence has showed a positive association related with coping with stressing situations [25, 26, 27], however, there seems to be a gap in literature regarding the association of emotional intelligence and the other discussed intelligences with the coming out process. The aim of this study was to examine if MIM can predict Coming Out Acceptance and Coming Out Growth among LGB Hispanic individuals living in Puerto Rico. The hypothesis of this pilot study was that the MIM will significantly predict the Coming Out Acceptance and Coming Out Growth with more than 10% of the variance. Validating this hypothesis provided us evidence to develop prevention and intervention programs to reduce health disparities associated with the coming out process among the LGB Hispanics Adults.

## Method

We implemented a quantitative pilot study with a cross-sectional approach and exploratory design. The study was approved by the Institutional Review Board (#2006039528) of the Ponce Health Sciences University.

### Participants

We used G*Power to compute the estimated minimum standardized effect size for the linear multiple regression. Assuming one tail, 0.30 effect size (medium), alpha error probability of .05, and a power of .95, a minimum sample size was 53. We employed a stratified sample procedure to ensure maximal variation in our sample, increase internal validity, and obtain the greatest comparison across the sexual orientations. A total of 67 participants (14 participants more than expected) completed the online study (13 lesbian women, 26 gay men, 15 bisexual women, and 13 bisexual men). Selection of these participants was based on convenient sampling following inclusion criteria: 1) being an adult between 21 to 39 years old, 2) identify as lesbian, gay, or bisexual, 3) identify as cisgender, 4) identify as Hispanic-Puerto Rican, 5) live in Puerto Rico, and 6) being out to at least one person. Since most of the population in the islands of Puerto Rico identify as Hispanic, and they are naturally born as United States of America (USA) citizens, Puerto Rico is an ideal place to conduct research about non-migrant Hispanics individuals. This condition allows researchers reduce additional associated with minority stressors due to the intersectionality of ethnic/race stigma and migratory status, that usually go along with Hispanics living in the continental USA [9].

The sample had an average of 28 years old. Also, 62.7% reported to have at least one partner, 71.6% were from an urban area, 4.5% informed a functional diversity, and 31.3% identified a religion or spiritual affiliation. Area of residence in the Island were also informed being 50.7% from the metropolitan area. Moreover, 40.3% reported an approximate income below $10,000 and other 43.3% between $10,001 and $30,000 (See Table 1).

### Instruments

#### Demographic Data Questionnaire

This questionnaire had 12 questions including age, sex identity (e.g., male, female, intersex, other), gender identity (e.g., feminine, masculine, other), sexual orientation, relationship, place and area of residence, income, functional diversity, religious affiliation, violence experiences, and coming out symptomatology.

#### Coming Out Acceptance Scale

This scale is composed of 10 items [28]. It was developed and preliminary validated by the team in this study. This scale has a Cronbach’s alpha of .72. Each participant had a list of people (father, mother, brother/sister, close friends, and other) for which they had to indicate their perception of acceptance level at the present. The ratings were a 5-point Likert scale (1 not accepted at all to 5 fully accepted, and 0 = does not apply).

#### Coming Out Growth Scale

This scale is composed of 35 items [2]. It was translated and culturally adapted into Spanish. This scale has a Cronbach’s alpha of .72 in the original validation. The participants could answer on a 5-points Likert scale (1 not at all to 5 a lot). This scale has two subscales, Individualistic Growth (IG) and Collectivistic Growth (CG).

#### Body Insight Scale

The Spanish version scale is composed of 36 items [18]. It was known by the name of Body Intelligence Scale. This scale has a Cronbach’s alpha of .89. The participants could answer on a 5-points Likert scale (1 strongly disagree to 5 strongly agree). This scale has three subscales: Energy Body Awareness, Comfort Body Awareness, and Inner Body Awareness.

#### Emotional Intelligence Scale

This scale is composed of 20 items [19]. This scale has a Cronbach’s alpha of .84. The participants could answer on a 4-point Likert scale (1 strongly disagree to 4 strongly agree). This scale has four subscales, Personal Recognition Competence, Personal Regulation or Management Competence, Social Recognition Competence, Social Regulation, or Management Competence.

#### Tromsø Social Intelligence Scale

This scale is composed of 21 items [20]. It was translated into Spanish. This scale has a Cronbach’s alpha of .79. The participants could answer on a 7-point Likert scale (1 strongly disagree to 7 strongly agree). This scale has three subscales Social Information Processing, Social Skills, and Social Awareness.

#### Ecological Intelligence Scale

This scale is composed of 12 items [21]. It was translated into Spanish. This scale has a Cronbach’s alpha of .82. The participants could answer on a 5-point Likert scale (1 completely disagree to 5 completely agree). This scale has three subscales: Holistic, Social Intelligence, and Economy.

### Recruitment And Procedure

We used a community outreach approach to recruit participants across the Island which gave us access to a diverse pool of possible participants. We also used Facebook ads to promote the study flyer. SurveyMonkey platform was used to access the anonymous survey. After accepting the platform informed consent, participants were able to complete sociodemographic and instrument data. Once they completed the survey, they were redirected to another REDCap platform page (to maintain confidentiality) where they gave their email information to receive a $25 gift card incentive for their participation. The gift cards were given to each participant who provided their email address.

## Results

Data were analyzed in the IBM SPSS Statistics Program (25.0v). First, we calculated the internal consistency indices (Cronbach’s Alpha), obtained the means and standard deviations of the measurements. Correlation analyses were performed between all study variables using Pearson’s Product-Moment Coefficient (r). To verify internal consistency, values were supposed to be greater than .70 [29]. To interpret the correlations, the classification suggested by Taylor [30] was used: correlations from .01 to .35 are considered low, between .36 and .67 moderate, between .68 and .89 high and, finally, .90 henceforth are considered very high. To identify the types of intelligence that could predict the scores of the dependent variables Coming Out Acceptance and Coming Out Growth, a series of multiple regressions were performed using the stepwise method. The effect size of the predictor on the predicted variable was established through the standardized regression Coefficient (β). Values of β lower than .09 were interpreted as a trivial effect size, between .10 and .29 small, between .30 and .49 medium, between .50 and .69 large, and greater than .70 very large [31]. All results were considered significant for p values <.05. Linear regression analyses were performed considering the entire sample and then by four stratified groups.

Analysis revealed that all the scales obtained acceptable values of internal consistency higher than .70. The results of the correlations show that the variable Coming Out Acceptance was not associated with any type of intelligence. However, the variable Coming Out Growth correlated between low and moderate with most types of intelligence, except for ecological intelligence. Table 2 shows the descriptions and correlations in detail.

### Coming Out Acceptance

A first linear regression analysis was carried out considering the entire sample to examine which variables (Body Intelligence, Emotional Intelligence, Social Intelligence, and Ecological Intelligence) best predict the Coming Out Acceptance. The model was not significant (*F* [4, 62] = 1.550, p = .199) at a significance level of .05 (see Table 3).

### Coming Out Growth

Subsequently, a linear regression analysis was performed considering the entire sample to examine which variables (Body Intelligence, Emotional Intelligence, Social Intelligence, and Ecological Intelligence) best predict the Coming Out Growth. The model was significant (*F* [1, 65] = 16,864, p < .001) and explained20.6% of the variance of the variable of Coming Out Growth. However, the only significant predictor in the model was the social intelligence variable (*β* = .45, p < .01) (see Table 3).

## Discussion

Preliminary findings of this pilot seem to indicate that the MIM initially predict the Coming Out Growth. On the contrary, preliminary findings showed no prediction for the Coming Out Acceptance. We believe this can be explained because in the Coming Out Acceptance process people may not have control of how other people can feel and react to the disclosure of their sexual orientation. On the other hand, in the Coming Out Growth process the person could have particular in fluence of their own growth [2]. This means that creating an intervention based on the model could help increasing the Coming Out Growth (e.g., developing a positive personal and social identity that is healthily shared with others), especially taken into consideration factors that interact with Hispanic LGB individuals such as familism, and *machismo* [9]. Moreover, social intelligence seems to be the more predictive factor in the model of the Coming Out Growth. However, the model requires further steps, including a larger sample to continue establishing its validating and to reduce the possibility of statistical errors.

It is too early to conclude on the findings of this model due to its pilot nature. However, preliminary findings suggest early evidence to continue working with the validation of this predictive model. The coming out is a diverse and ongoing process, and its results can vary each time. Thus, it is crucial and beneficial to provide LGB individuals preparation and coping skills such as fluid intelligences to better manage this process and prevent any potential effects on their physical, psychological, social, and environmental health consequences. Specially, when this process is followed by rejection or tolerance. Body intelligence can help the individual to be aware of their physical symptoms (e.g., sweating, elevated palpitations, tremors) when planning or conducting the coming out, preventing and/or handling subsequent physical health consequences (e.g., fainting, dizziness, chest pain). Similarly, emotional intelligence can help the individual to be aware of their psychological symptoms (e.g., anxiety, stress, depression) and improve their ability to manage and organize their feelings and emotions, before and after the coming out process, in order to better oversee their thoughts and actions.

Moreover, social intelligence can increase the ability of social and interpersonal awareness before, during, and after the coming out process by increasing the ability to understand information like safety, importance, and the context of the potential reaction. At the same time, being prepare for possible unhealthy, adverse, or dangerous scenarios (e.g., rejection, violence, homelessness). Finally, ecological intelligence helps the individual dealing more effectively with the cultural, systematic, and environmental components of the coming out process.

Strengths and limitations were also present in this study. Some of the strengths of the study are: 1) the proposed sample was achieved, 2) sex and sexual orientation was stratified following the guidelines of the National Institutes of Health, and 3) internal consistency of the instruments were adequate. On the other hand, some limitations are also present: 1) the Coming Out Acceptance Scale was constructed and preliminarily validated for this study, despite, acceptable values of internal consistency were obtained, 2) all the other instruments were translated, culturally adapted, and preliminarily acceptable values of internal consistency were obtained for this study, but a larger sample is needed for a formal internal consistency validation, and 3) a medium effect size sample was used for this study which affects the statistical power.

With the successful validation of this model our team can develop prevention and intervention programs to reduce health disparities among the LGB Hispanic community due to the coming out process. This innovative model created by and for Hispanics, can also be adapted in future studies from different disciplines to reduce psychosocial disparities in other vulnerable minority groups. Future research directions can include the use of the model for other concerns inside and outside sexual minorities, the use of the model with other developmental stages (e.g., adolescents, older adults), and the use of the model with other ethnic/race groups. The coming out instruments can also be adapted to be used with gender minorities. Furthermore, other types of fluid intelligences can be integrated in the model (e.g., spiritual intelligence).

## Figures and Tables

**Table 1:** Demographic Characteristics of the Sample

Characteristics	*n*	*%*
Sex Identity		
Female	28	41.8
Male	39	58.2
Sexual Orientation		
Bisexual	28	41.8
Lesbian	13	19.4
Gay	26	38.8
Age		
21–25	19	28.3
26–30	25	37.3
31–35	19	28.3
36–39	4	6
Partner/s		
Yes	42	62.7
No	25	37.3
Geographical Area		
Urban	48	71.6
Rural	19	28.4
Geographical Location		
Metropolitan (capital)	34	50.7
North	8	11.9
South	4	6.0
East	6	9.0
West	9	13.4
Central		9.0
Functional Diversity		
Yes	3	4.5
No	64	95.5
Individual Annual Income		
Less than 10,000	27	40.3
10,001–30,000	29	43.3
30,001–50,000	6	9.0
50,001–70,000	2	3.0
More than 70,001	3	4.5
Religious Affiliation		
Yes	21	31.3
No	46	68.7

**Table 2:** Correlations Between the Variables, Means, Standard Deviations and Cronbach’s Alpha

Instruments	*α*	*M*	*DE*	1	2	3	4	5
1. Coming Out Acceptance	.72	4.36	1.21	-				
2. Coming Out Growth	.95	117.75	23.89	.50**	-			
3. Body Intelligence	−76	70.00	7.90	.20	.36**	-		
4. Emotional Intelligence	.86	70.06	6.10	.03	.30*	.52**	-	
5. Social Intelligence	.85	97.43	17.75	.17	.45**	.55**	.33**	-
6. Ecological Intelligence	.84	49.64	7.83	.19	.15	.20	.34**	.08

Note.

** = *p*<.01;

*= *p*<.05;

*α* = Cronbach’s Alpha. (*n*=67).

**Table 3:** Regression Coefficients and Associations Between Coming Out and Intelligences

Variables	*B*	*SE*	*β*	*t*	*p*	95% CI
Coming Out Acceptance^*a*^ *[R* = .302; *R*^*2*^ = .091; Δ*R*^*2*^ = 1.186; *F*= 1.550]						
Constant	2.414	1.792		1.347	.183	[-1.169,5.996]
Body Intelligence	.030	.025	.199	1.238	.220	[-.019, .080]
Emotional Intelligence	−.034	.029	−.174	−1.170	.246	[-.093, .024]
Social Intelligence	.007	.010	.104	.714	.478	[-.013, .027]
Ecological Intelligence	.031	.020	.201	1.557	.125	[-.009, .071]
Coming Out Growth^a^ *[R* = .488; *R*^*2*^ = .238; Δ*R*^*2*^= .189; *F=* 4.849**]						
Constant	11.954	32.495		.368	.714	[-53.002,76.911]
Body Intelligence	.257	.445	.085	.578	.565	[-.633,1.148]
Emotional Intelligence	.431	.532	.110	.809	.421	[-.633,1.495]
Social Intelligence	.492	.179	.366	2.745	.008**	[.134, .850]
Ecological Intelligence	.195	.360	.064	.541	.591	[-.525, .915]

Note.

** = *p*< .01;

* = *p*< .05.

(*n* = 67).

## Data Availability

The datasets used and/or analyzed during the current study are available from the corresponding author on reasonable request.
